# Neuroprotective activity of green synthesized silver nanoparticles against methamphetamine-induced cell death in human neuroblastoma SH-SY5Y cells

**DOI:** 10.1038/s41598-023-37917-0

**Published:** 2023-07-22

**Authors:** Sadegh Khorrami, Manijeh Dogani, Saeed Esmaeili Mahani, Mehrdad Moosazadeh Moghaddam, Ramezan Ali Taheri

**Affiliations:** 1 Nanobiotechnology Research Center, Baqiyatallah University of Medical Sciences, Tehran, Iran; 2 Department of Biology, Faculty of Sciences, Shahid Bahonar University of Kerman, Kerman, Iran; 3 Tissue Engineering and Regenerative Medicine Research Center, Baqiyatallah University of Medical Sciences, Tehran, Iran

**Keywords:** Biomaterials, Nanobiotechnology, Neurology

## Abstract

The present study aimed to investigate the neuroprotective activity of the black peel pomegranate extract, and silver nanoparticles (AgNPs) biosynthesized using the extract. We pretreated the human neuroblastoma SH-SY5 cells with the extract and AgNPs and evaluated the neuroprotective activity of these agents against methamphetamine (Meth) cytotoxicity. The NPs were spherical with 19 ± 8 nm size, − 28 mV surface charge, and 0.20 PDI. Meth killed the cells by increasing proapoptotic (*Bax*, *PTEN*, *AKT*, *PI3K*, *NF-κB*, *P53*, *TNF-α*, *Cyt C*, and *Cas 3*) and decreasing the antiapoptotic genes (*Bcl-2*) expression. Exposure to Meth caused DNA fragmentation and increased the intercellular ROS and malondialdehyde (MDA) levels while reducing the mitochondrial membrane potential (MMP). A 4-h pretreatment of the cells with the extract and AgNPs could retain the viability of the cells above 80% by increasing the *Bcl-2* expression up to fourfold and inhibiting the cell death pathways. ROS, MDA, and MMP levels in the pretreated cells were close to the control group. The percentage of necrosis in cells pretreated with the extract and AgNPs declined to 32% and 8%, respectively. Our promising findings indicated that AgNPs could reduce Meth-induced oxidative stress and prevent necrotic and apoptotic cell death by regulating related genes’ expression.

## Introduction

Approximately 25 million people use amphetamine-type stimulants, especially methamphetamine (Meth), all around the world, which is more than those who abuse heroin and cocaine combined^1^. Due to its low cost, low production cost, and long duration of action, the drug is highly sought-after by abusers^2^, while it has been established that Meth abuse can lead to serious health complications in humans^3^. Besides causing neurodegenerative subsequences in the brains of addicts, this drug can also damage other body organs’ functions^4^. Symptoms associated with this substance include acute agitation, anxiety, aggressive behaviors, paranoia, hypertension, hyperthermia, and psychosis^5^.

As recently revealed, the drug can induce apoptosis in the nervus system, resulting in the death of neuronal bodies^6^. The mechanism behind the toxic effects of Meth is linked to its dopamine-like chemical structure, allowing it to penetrate dopamine axons, release dopamine from cytoplasmic vesicles, and transport it into the synaptic clefs^7^. The interaction between dopamine quinones and superoxide radicals in nerve terminals results in Meth neurotoxicity. In addition to hydrogen peroxide production, dopamine metabolism can raise toxic hydroxyl radicals, especially when interacting with metal ions^8^. The accumulation of evidence suggests that Meth also causes oxidative stress by causing an imbalance between the production of reactive oxygen species (ROS) and the ability of antioxidant enzymes to scavenge them. Overproduction of ROS can damage mitochondrial and nuclear DNA, lipids, and proteins in cells^9,10^.

Some approaches have been proposed to protect the neurons against Meth toxicity. Recently, it has been revealed that methamphetamine-associated toxicity is attenuated by antioxidants, lipid peroxidation inhibitors, and spin-trapping compounds, which inactivate free radicals^11^. In this way, enhancing the expression of the antioxidant enzymes, as well as applying external antioxidant compounds, can be beneficial in protecting against methamphetamine-induced neurotoxicity. In this regard, phytocompounds have shown to be excellent antioxidants^12–15^, offering a great potential to enhance/assist the cellular antioxidant system and reduce the adverse impacts of cytotoxic compounds. In addition to plant extracts, the metal nanoparticles synthesized by mediating these extracts (green/bio-synthesized nanoparticles) may possess a high potential to protect the cells. There has been a great deal of evidence that these nanoparticles can go much farther than we imagined. The green synthesized nanomaterials are relatively cost-effective and eco-friendly because in their synthesis process, the metal salts are reduced by the extracts reducing agents, and the nanoparticles are coated with the extracts' bioactive compounds. Therefore, they inherit the extracts’ pharmaceutical properties and nanomaterial characteristics, such as large surface areas, tiny sizes, and high surface energy^16^.

Silver nanoparticles (AgNPs) are among the world's most popular metal nanoparticles owing to their biological activities and unique electronic and optical features^17,18^, arising from their collective oscillations of conduction electrons known as surface plasmon resonance^19^. Nowadays, many of the bio-medical properties of biosynthesized Ag nanoparticles are well-described, such as their antimicrobial, anticancer, antioxidant, and anti-inflammatory activities^20,21^. However, there is no report about their protective activity against cytotoxic agents. It seems that the defensive potential of the green synthesized nanoparticles has been overlooked up to now.

We hypothesized that silver nanoparticles synthesized using the black peel pomegranate extract would be a neuroprotective agent against the cell death induced by Meth in the SH-SY5Y cells (Human Neuroblastoma Cell Line). To test this hypothesis, we pretreated the cells with the extract and nanoparticles, followed by exposing them to Meth. A comparison of the viability of the pretreated cells and non-pretreated ones indicated that these compounds could significantly defend the cells by regulating the genes involved in cell death (apoptosis and necrosis). These protective agents could also inhibit the cells' DNA fragmentation, decrease the intercellular ROS levels, reduce the MDA levels, and maintain the mitochondrial membrane potential at normal levels.

## Results

### Physicochemical characterization

#### UV–visible

**Figure 1:**
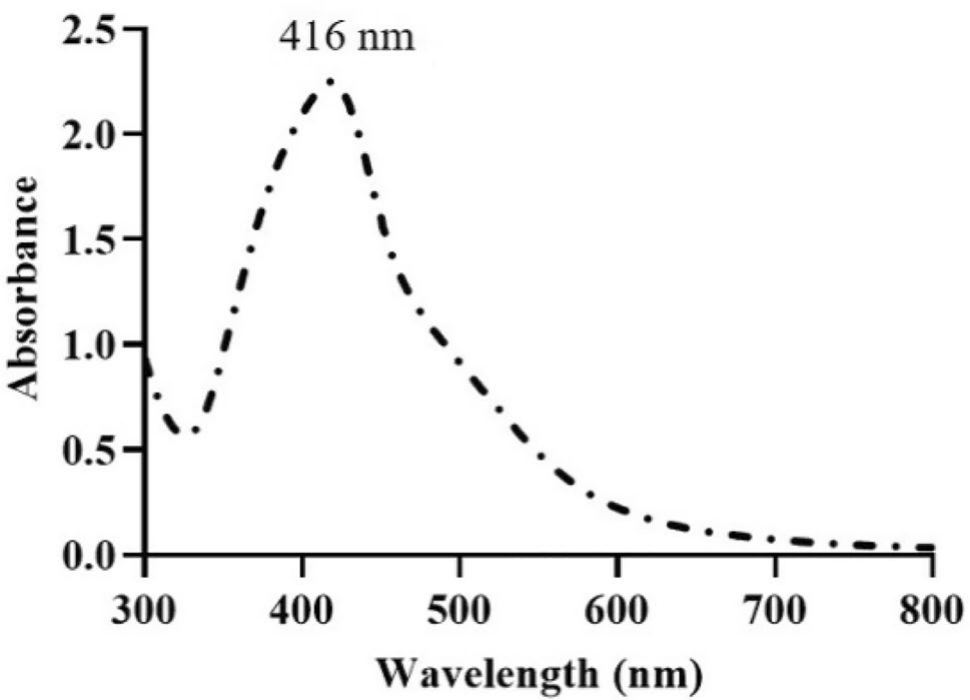
The UV–visible spectrum of the green synthesized AgNPs using black peel pomegranate extract.

Based on the primary analysis, the mixture of the extract and AgNO_3_ resulted in a dark-red colloid of AgNPs, which were entirely stable for more than 6 months in the lab environment (this observation was confirmed by zeta potential analysis too). Also, a single sharp absorbance peak at 416 nm appeared in the UV–Vis spectrum of the colloid (Fig. 1). The dark-red color of the final mixture, as well as the peak that appeared at 416 nm, are attributed to the changes in the metal nanoparticles’ surface plasmon resonance (SPR). These features are known as the characteristics of AgNPs colloid and confirm the success of the biosynthesis process^22^.

#### FTIR

**Figure 2:**
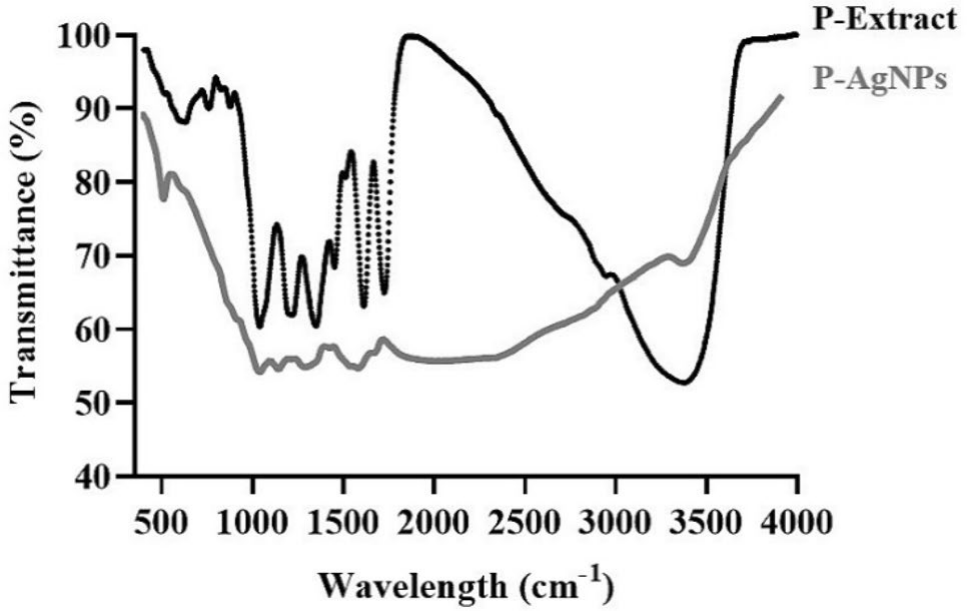
FTIR spectra of the black peel pomegranate and AgNPs synthesized using the extract.

A comparison of the FTIR spectra of the extract and AgNPs is shown in Fig. 2. The spectrum pattern of these two compounds was nearly identical, with a distinctive peak at 3450–3550 cm^−1^ associated with the OH group. Also, in these spectra, shoulder peaks for C–C, N–H, and C–O functional groups could be observed between 1100 and 1700 cm^−1^. However, in the spectrum of nanoparticles, the OH-associated peak was weaker than in the extract, which can be due to the involvement of the OH groups in the reduction of Ag^+^ to Ag^0^^23–25^.

#### DLS and Zeta

**Figure 3:**
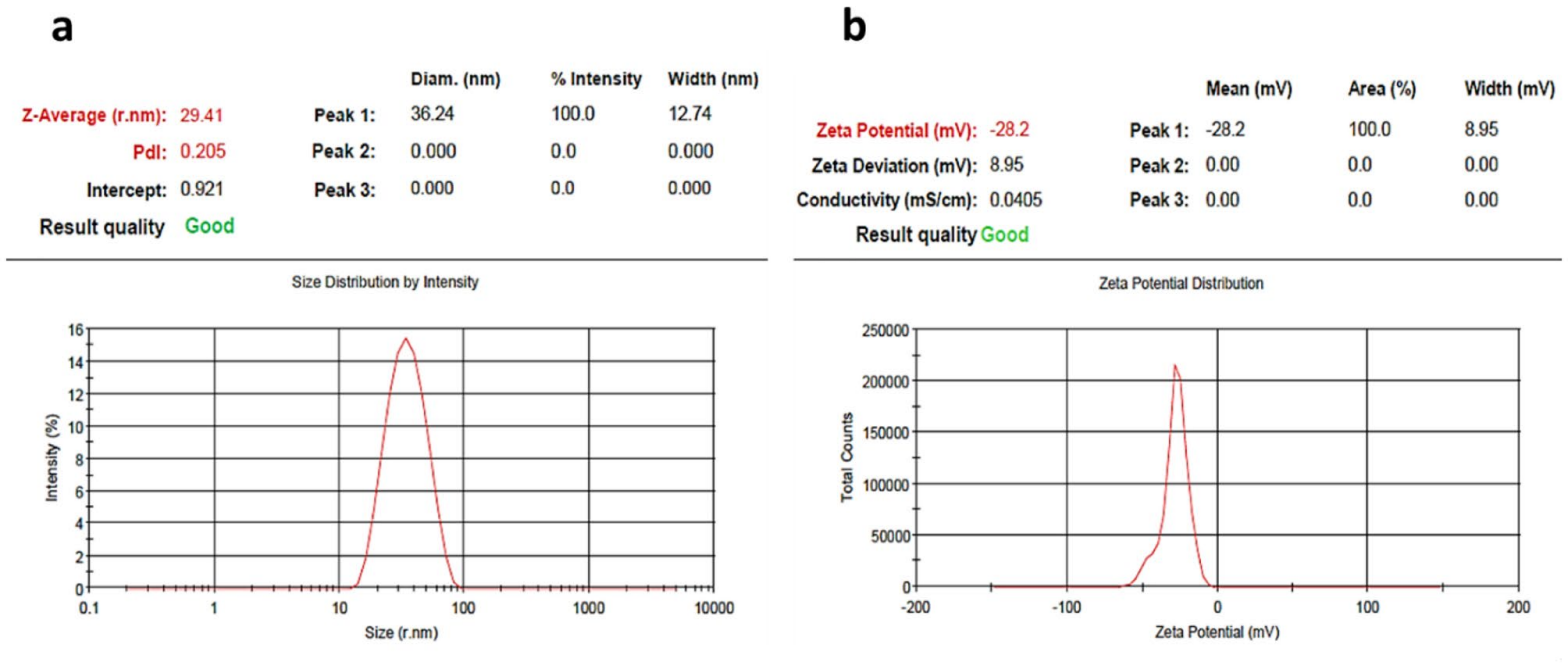
DLS (**a**) and Zeta potential (**b**) of AgNPs green synthesized using black peel pomegranate extract.

Figure 3 shows the DLS and Zeta potential analysis of the biosynthesized nanoparticles. Based on the results, these nanoparticles were at 29.5 nm average size with the polydispersity index (PDI) of 0.20 and − 28.2 mV surface charge. These features altogether are responsible for the desirable properties of the nanoparticles' monodispersity, and stability. As previously determined, a PDI lower than 0.5 means monodispersity, and a PDI higher than 0.75 means polydispersity^26,27^. Also, a high surface charge (+ or −) results in a strong repulsive force between nanoparticles, making their colloids stable. Hence, the nanoparticles' low zeta potential can justify their colloid's stability.

#### TEM

**Figure 4:**
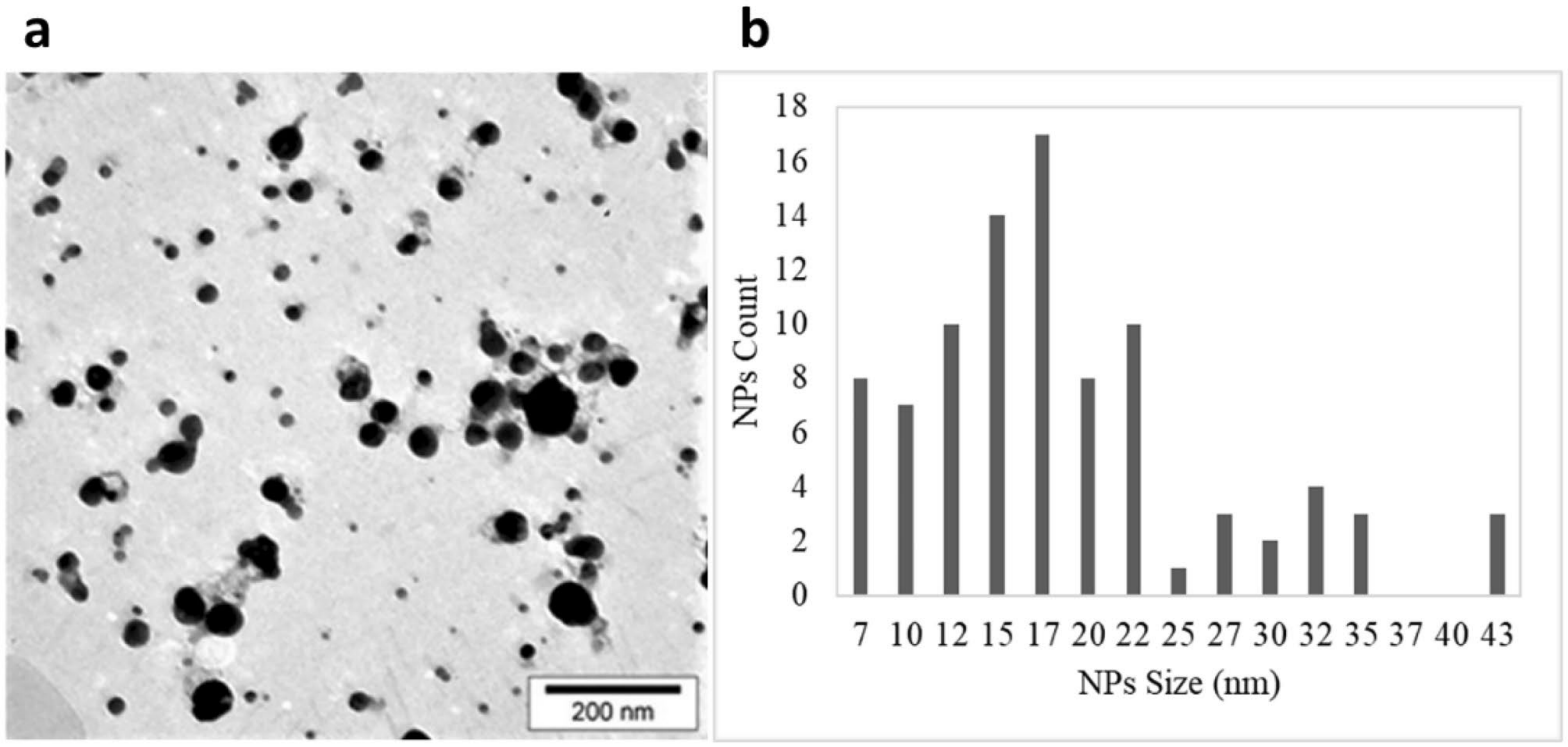
The TEM image of biosynthesized silver nanoparticles (**a**) and their size distribution diagram (**b**).

Transmission Electron Microscopy (TEM) analysis revealed the morphology and size of Ag nanoparticles (Fig. 4). As results showed, the particles were spherical in shape with 19 ± 8 nm of size and adequate dispersity. These results were in line with the DLS and zeta analyses results. Though, the DLS showed a slightly larger average size, which can be related to the hydrodynamic size of nanoparticles.

### Cytotoxicity assay

**Figure 5:**
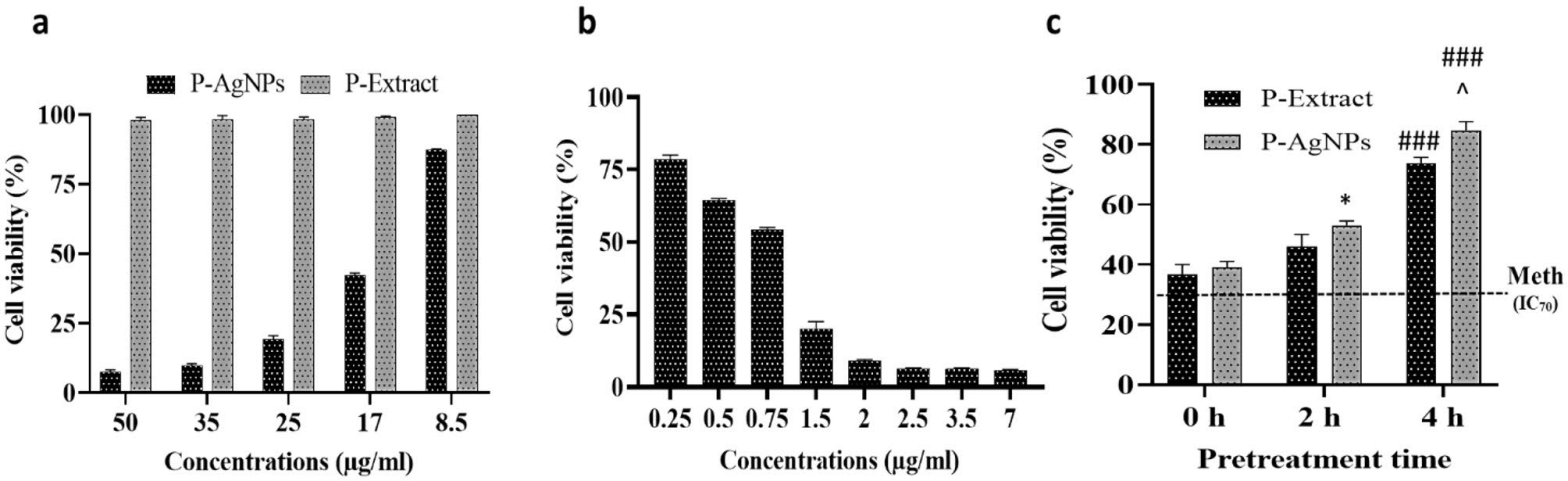
(**a**) The cytotoxicity of different concentrations of green synthesized nanoparticles (P-AgNPs) and the extract (P-Extract) on SH-SY5 cells. (**b**) The cytotoxicity of different concentrations of methamphetamine on SH-SY5 cells. (**c**) The viability of cells exposed to methamphetamine for 24 h after 0, 2, and 4 h of pretreatment with P-extract and P-AgNPs. Data are expressed as mean ± SEM. ^*^P < 0.05 compared to 0 h; ^###^P < 0.001 compared to 2 h; ^^^P < 0.05 compared to P-Extract.

Firstly, the MTT assay was applied to investigate the toxicity of different concentrations of the extract, Ag nanoparticles, and Meth on the SH-SY5 cells. It was indicated that the extract was innocent to the cells at all tested concentrations. Likewise, low concentrations of nanoparticles did not show a considerable effect on the cells. Figure 5a shows that 8.5 µg/ml of this compound resulted in only 20% cell death. The lower concentrations were totally safe for the cells (data not shown). Though, the cytotoxicity of Meth toward the cells was substantial so that 1.5 µg/ml of this compound could induce more than 75% of cell death (Fig. 5b).

### Protective effect

#### Cell survival

To determine whether the extract and nanoparticles could protect the cells against Meth or not, the cells were treated with these agents for 0 h, 2 h, and 4 h before exposing them to the IC_70_ concentration of Meth. The comparison between the viability of these 3 pretreated groups and non-pretreated ones showed that the compounds could markedly protect the cells in a time-dependent manner (Fig. 5c). That is, the protective effect increases with increasing pretreatment time. Regarding the cells subjected to the extract/AgNPs immediately before exposure to Meth (0 h pretreatment), the cells’ survival was about 40%. Yet, this feature rose to approximately 85% after 4 h of pretreatment. The viability of non-treated cells was about 30%.

#### ROS assay

**Figure 6:**
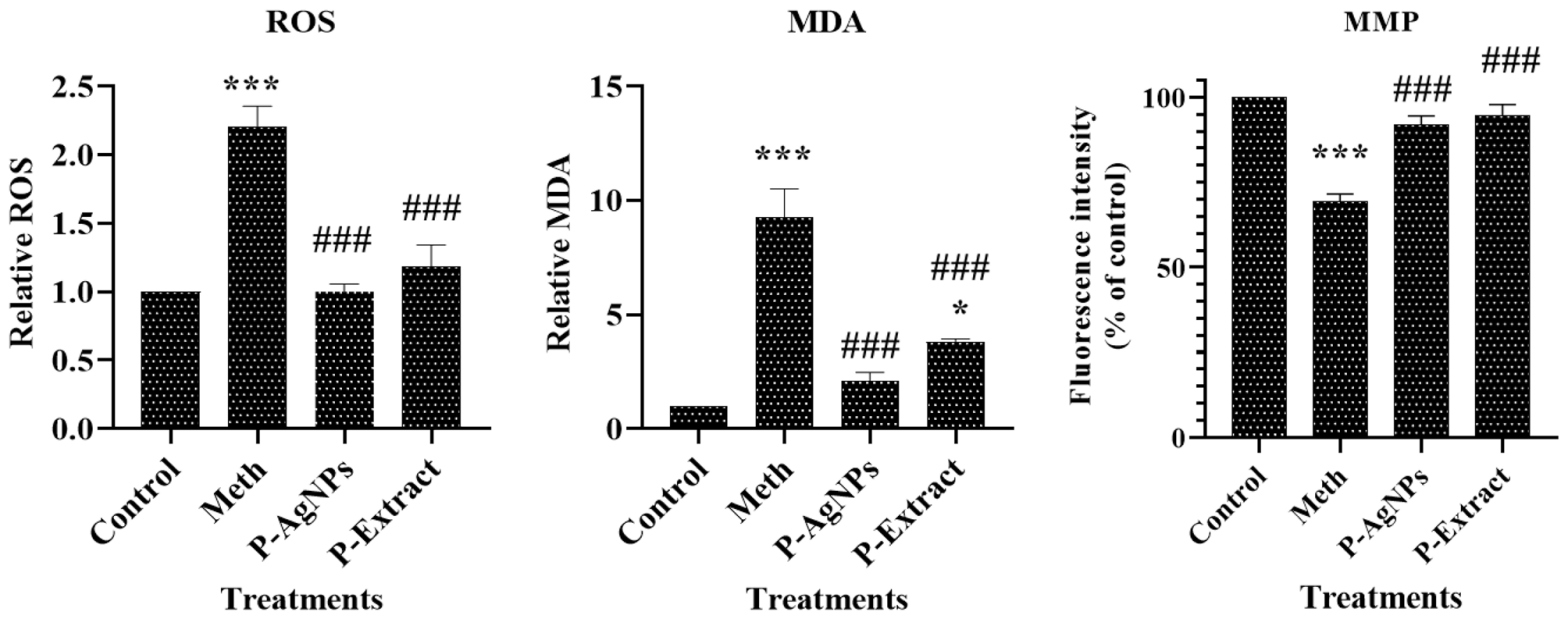
The intracellular ROS levels (ROS), Malondialdehyde (MDA) levels, and the mitochondrial membrane potential (MMP) of SH-SY5 cells exposed to methamphetamine. The charts compare these features in the cells pretreated with P-AgNPs, and P-Extract and none-pretreated ones. Data are expressed as mean ± SEM. *P < 0.05, and ***P < 0.001 compared to control; ^###^P < 0.01 compared to Meth.

As Fig. 6 shows, the amount of intracellular ROS increased more than twofold in the presence of Meth. On the other hand, 4 h pretreatment of the cells with the extract and nanoparticles could reduce the ROS levels to 1.1-fold. The reducing effect of the extract was more than AgNPs, probably due to its higher antioxidant activity^28^.

### Malondialdehyde (MDA) assay

Thiobarbituric acid reactive substance (TBARS) assay is a method to detect lipid oxidation. The purpose of this assay is to measure the malondialdehyde (MDA) level, which is formed when unsaturated fatty acids are oxidized. Based on the results, MDA levels in the cells treated with Meth substantially increased (up to eightfold). Pretreatment of the cells with the extract and AgNPs, however, reduced this level to about two and fourfold of the control, respectively (Fig. 6).

### Mitochondrial membrane potential assay (MMP)

The MMP was quantitatively measured in Meth-subjected cells pretreated with the extract and AgNPs in comparison with non-pretreated ones. According to the results (Fig. 6), ΔΨm value did not show considerable change in pre-treated cells, whereas this feature dropped by 30% in the cells exposed to the Meth.

### DNA fragmentation

**Figure 7:**
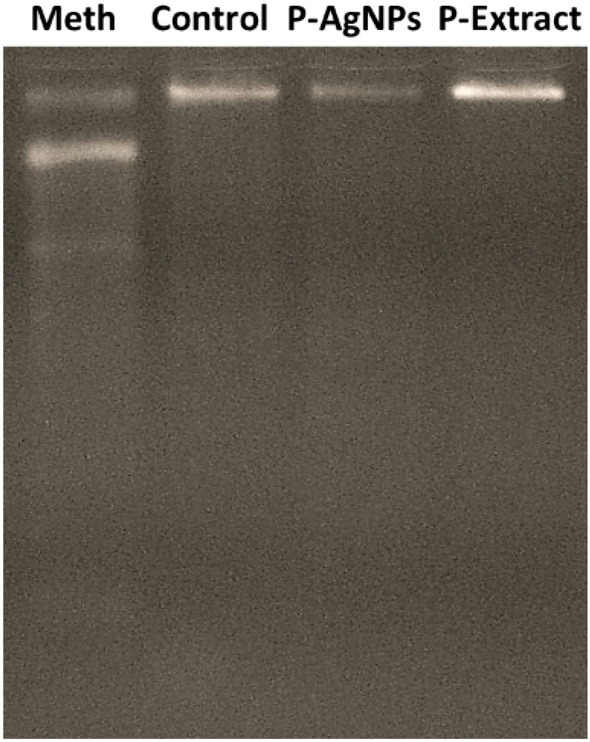
The agarose gel shows the DNA segments of SH-SY5 cells exposed to Methamphetamine (Meth), green synthesized nanoparticles (P-AgNPs), and pomegranate extract (P-Extract).

It was revealed that exposure to Meth could cut down the cells' DNA into 2–3 fragments. Nevertheless, pretreatment of the cells with the extract and AgNPs could prevent DNA fragmentation. As Fig. 7 shows, concerning the Meth-treated cells, several bands (smear-like) formed on the agarose gel, while only a single sharp band was observed in the pretreated cells (Supplementary Information).

### Gene expression assay

**Figure 8:**
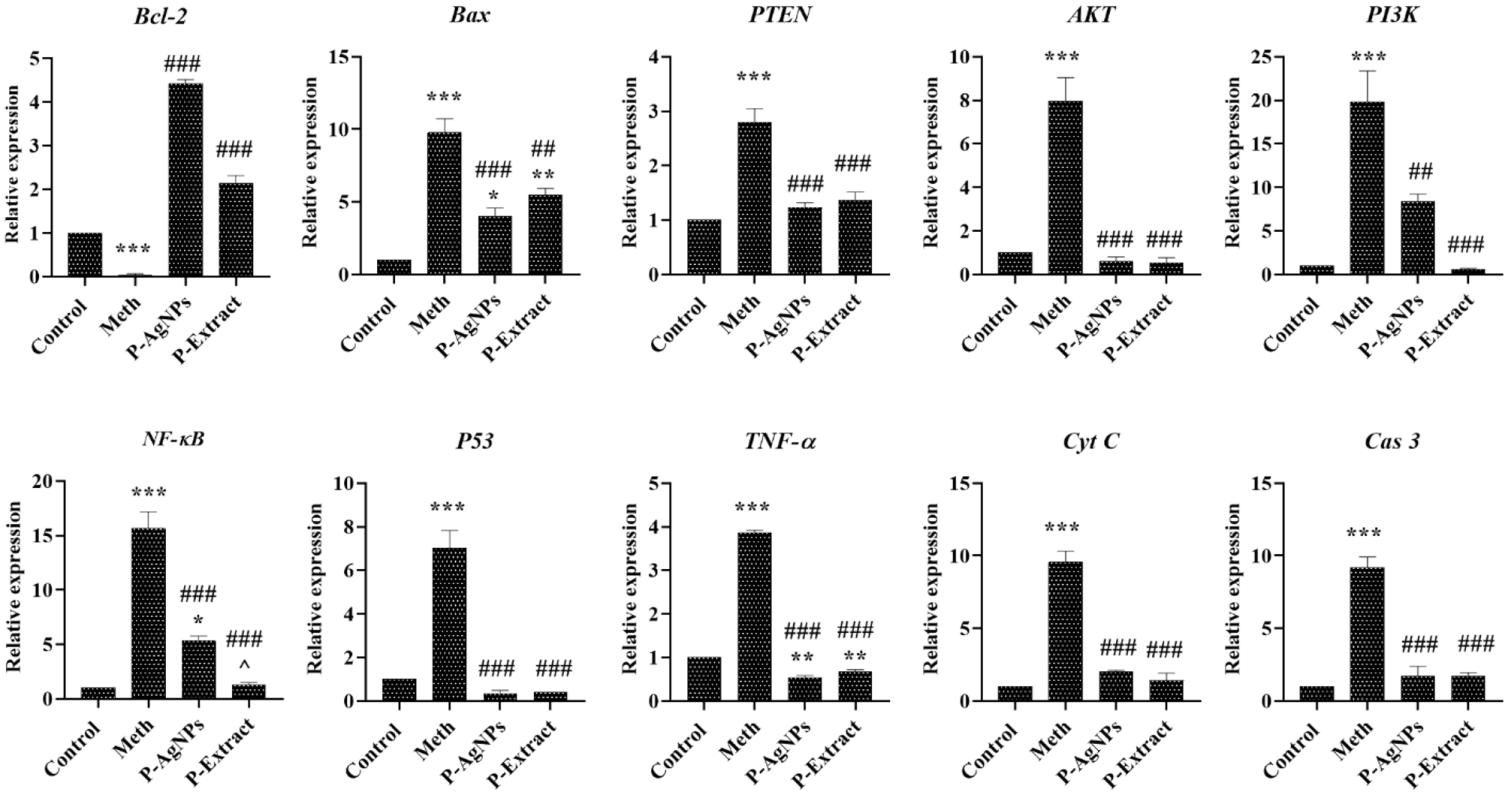
The RT-PCR results show the expression of different key genes involved in the cell death process in the cells pretreated with the extract and nanoparticles and non-pretreated cells. Data are expressed as mean ± SEM. *P < 0.05, **P < 0.01 ,and ***P < 0.001 compared to control; ^##^P < 0.01,and ^###^P < 0.001 compared to Meth; ^^^P < 0.05 compared to P-AgNPs.

In order to determine the pathway(s) through which these agents induce their cytotoxic or protective effects, the expressions of 10 essential genes involved in the cell death/growth pathways were evaluated using RT-PCR. In line with MTT assay results, our findings indicated that in the cells exposed to the Meth, the expression of genes inducing cell death, such as *Bax*, *PTEN*, *AKT*, *PI*_*3*_*K*, *NF-κB*, *P53*, *TNF-α*, *Cyt C*, and *Cas 3*, considerably increased. In contrast, the expression of the genes supports the viability of cells, such as *Bcl-2*, degreased. However, pretreatment of the cells with the extract and AgNPs could significantly maintain the expression of the cell death-inducing genes at low levels (Fig. 8). Given that most of these genes are directly involved in the intrinsic and extrinsic pathways of apoptosis, it seems these protective agents through regulating the genes' expression against apoptosis protect the cells.

### Flow cytometry analysis

**Figure 9:**
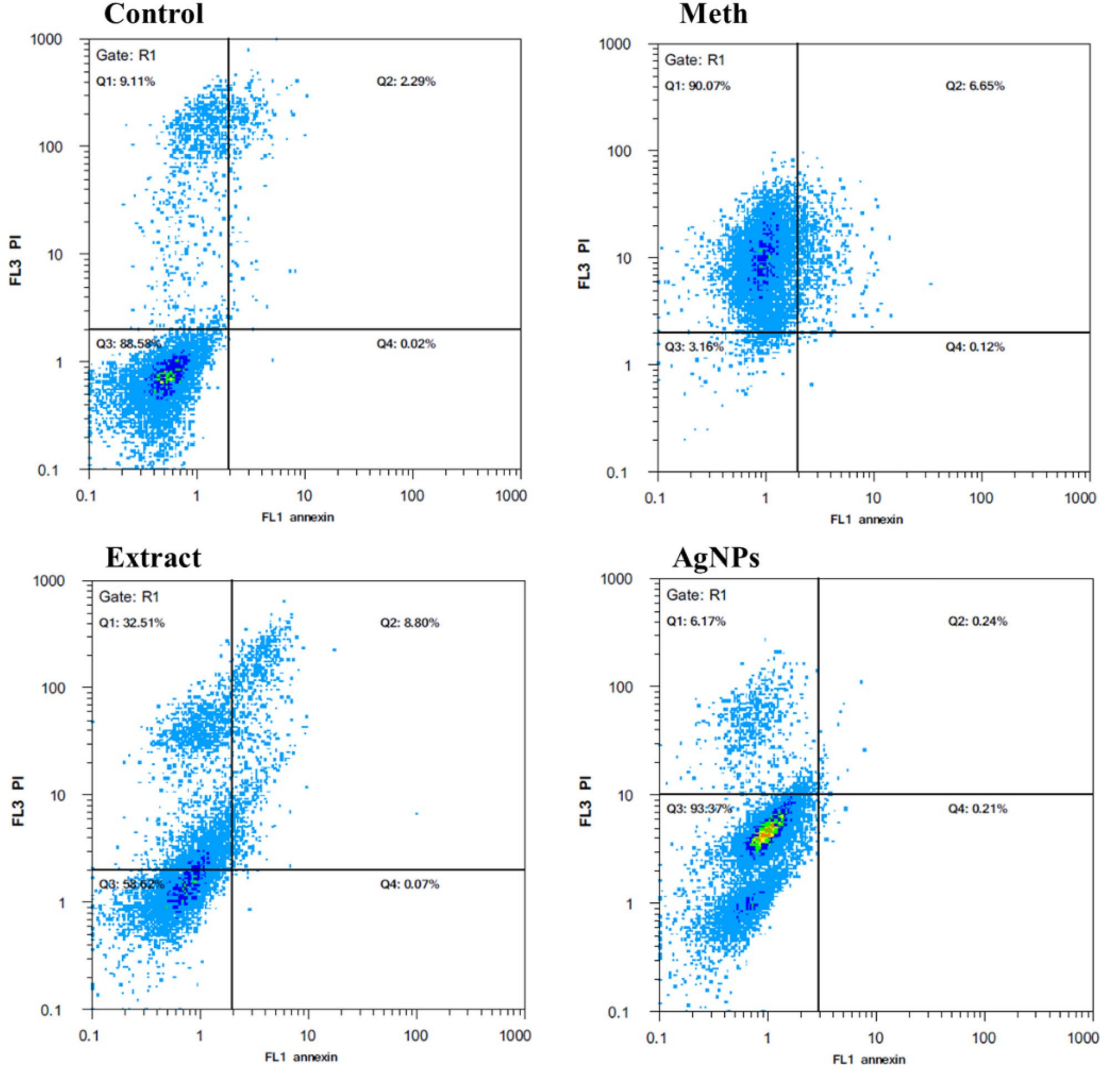
Flow cytometry analysis of the cells pretreated with the extract or AgNPs compared to non-pretreated cells.

Flow cytometry analysis revealed that the rate of death in the cells exposed to Meth was considerably high. Twenty-four hours of exposure to the drug caused 90% necrosis and 7% apoptosis in the cells. This is when the necrotic death in the cells pretreated with the extract or AgNPs increased to 33% and 6%, respectively. In addition, about 8% apoptosis was detected in the extract-pretreated cells, while no significant apoptosis was observed in the cells pretreated with AgNPs (Fig. 9).

## Discussion

The use of methamphetamine has reached epidemic levels and has become a rapidly growing global health issue due to its addictive properties. Unfortunately, there is no conventional pharmacotherapy for treating abusers/patients till now^29^. Among the most severe health complications associated with Meth addiction are deficiencies in attention, memory, and executive function. Besides affecting the nervous system, methamphetamine has also cardiotoxic and immunosuppressive effects^30^. It has been reported that these neuropsychiatric complications are caused by drug-induced neurotoxic effects, which include damage to dopaminergic and serotonergic terminals, induction of neuronal apoptosis, and activation of astroglial and microglial cells^6^. In this study, we evaluated the neuroprotective activity of the black peel pomegranate extract and the silver nanoparticles green synthesized using the extract. Our findings indicated that these agents have prominent protective activity against Meth-induced cell damage.

Based on our results, pretreatment of the cells could increase the *Bcl-2* gene expression while decreasing the expression of the *Bax* gene. In line with these findings, Cadet et al. have previously reported that the Bcl-2 family of proteins is involved in the mechanisms underlying Meth neurotoxicity^31^. In particular, Meth increases proapoptotic proteins, such as BAX, and decreases antiapoptotic proteins, like Bcl-2. Likewise, we found that the expression of Cyt c significantly increased in the presence of Meth. The upregulation of proapoptotic proteins is consistent with the findings that Meth treatment caused the release of mitochondrial Cyt c protein and apoptosis-inducing factors into the cytosol^32,33^. A recent study by Salari et al. reported an increased release of Cyt c from mitochondria and an increased Bax to Bcl-2 ratio in the presence of Meth^34^. The factors released from mitochondria have been shown to participate in Meth-induced apoptosis by activating Cas9 and 3 and breaking down several structural cellular proteins^35^. In this study, we observed the overexpression of the Cas 3 gene and Cyt c in the Meth-exposed cells, which is in agreement with the mentioned process. Thus, these findings implicate the mitochondrial pathway in Meth-related neuronal cell death.

It is becoming increasingly evident that mitochondrial dysfunction is one of the most important etiological factors of neurological disorders. The aging process and oxidative stress are two other common causes of neurological disorders. On the other hand, the incidence of apoptosis is a hallmark of neurodegenerative diseases, and its process can mainly be regulated by mitochondria function^36^. Hence, it could be concluded that the altered signaling of apoptotic mechanisms is involved in neurodegeneration.

We also investigate the genes mediating the PI3K/Akt/mTOR signaling pathway. This is an intracellular pathway required for cell cycle, proliferation, cell death, and autophagy and can be activated by various cellular stimuli and toxins. In previous studies, PI3K has been shown to transmit survival signals, which in turn trigger the phosphorylation of downstream molecules such as Akt^37^. Researchers have discovered that Akt regulates pro-apoptotic protein expression by triggering another protein kinase as well as transcription factors to promote survival signals^38,39^. Likewise, we observed the decreased expression of genes involved in this pathway in the cells treated with Meth. Furthermore, in our study, Meth could enhance the *NF-κB, P53,* and *TNF-α* gene expression. Similarly, Tan et al. reported that compared to the control group, treatment with Meth significantly increased the expression of *PI3K*, *Akt*, *PTEN*, *p53, Bax,* and *cas 3*^40^*.* Also, it has been revealed that exposure to Meth increased the expression of the *TNF*-α gene^41^.

Increasing the cellular ROS and MDA production, reduction of MMP levels, and fragmentation of DNA were other phenomena we observed in the cells exposed to Meth. These findings are related to gene expression changes. Interestingly, we found that pretreating the cells with the extract or NPs could significantly diminish the Meth detrimental effects on the cells and maintain the cells’ survival approximately at the control cells.

Mitochondria are also involved in producing ROS. Increased ROS levels initiate a cascade leading to apoptotic and non-apoptotic cell death^42^. Furthermore, ROS produced in cells acts as signaling molecules. Still, its overproduction will affect DNA, proteins, and lipids. It can also decrease the effectiveness of cellular mechanisms, initiate inflammatory pathways, cause excitotoxicity, agglomerate proteins, and trigger apoptosis^43,44^.

Mitochondrial membrane potential (ΔΨm) generated by proton pumps (complexes I, III, and IV) is an essential component in the energy storage process during oxidative phosphorylation. A long-term imbalance in ΔΨm levels may cause an unwanted loss of cell viability and cause various detrimental effects^45^. Additionally, it has already been revealed that oxidative stress induced by amphetamines increases lipids peroxidation, which in turn increases the concentration of MDA in the brain^46^ and causes DNA and cell membrane damage^47^. Accordingly, the increase in MDA level and DNA fragmentation of cells treated with methamphetamine can be justified.

Besides, we found that AgNPs provide cell protection at much lower concentrations than the extract. The defensive action of the extract and AgNPs can be attributed to their high antioxidant capacity. Based on recent studies, the black peel pomegranate extract and silver nanoparticles synthesized using the extract showed high antioxidant activity, owing to containing high levels of anthocyanin^28,48^. Anthocyanins, from the subclass of flavonoids, are a significant part of phenolic compounds and form red, blue, and purple pigments in plants^49^. Noteworthy, there is evidence showing the cytotoxicity of green synthesized silver nanoparticles against SH-SY5 cells^50^, though our observations indicated the harmlessness of the nanoparticles toward the cells, especially at low concentrations.

Our findings support the argument that a mitochondrial pathway is most likely involved in the Meth-induced cell death. Arguably, this pathway is considerably inhibited in the cells pretreated with pomegranate extract and the green synthesized Ag nanoparticles. Proposing a precise mechanism of action justifying the neuroprotective activity of these agents needs further studies. However, we believe that the pathways through which the extract and AgNPs induce their effect are not limited. The nanoparticles are tiny and covered by phytochemicals, whereby are efficiently uptake by the cells. Subsequently, they not only pursue their function as an external antioxidant source but also trigger the cells' antioxidant systems and mediate the up-regulation of the expressions of the genes responsible for maintaining the cells' survival. Whereby, they can alert the cells' defense system, enhancing the ability of cells to overcome the damage caused by Meth.

## Conclusion

Herein, we evaluated the neuroprotective activity of the black peel pomegranate extract and silver nanoparticles green synthesized by mediating the extract. Our findings revealed that pretreatment of the neural cells with both compounds alleviates the toxic effects of Meth, though the administration of AgNPs was considerably more advantageous. These agents regulate the expression of antiapoptotic and proapoptotic genes in favor of cell survival. They also reduced the cellular ROS and MDA levels and prevented the MMP reduction in the cells exposed to the Meth. Taking this data into account, we can draw the researchers' attention to green synthesized nanoparticles' neuroprotective potential. Additional in vivo and clinical studies are needed for a pharmaceutical formulation applying the biosynthesized nanoparticles and the pomegranate extract in a practical treatment alleviating the psycho-neurological complications of patients with Meth addiction.

## Methods

### Preparation of the extract

The black peel pomegranate (*Punica granatum*) samples were purchased from a local gardener located in Darab city, Fars province, Iran. The fruits were transferred to the lab, washed with distilled water, and peeled up. Next, 100 g of the peels were soaked in 300 ml of deionized water and shaken using a shaker-incubator for 24 h at 40 °C. Then, a Whatman filter paper (grade 1) was utilized to filter the mixture, followed by concentrating the filtrate with a rotary evaporator (EYELA N-1100, Tokyo Rikakikai Co. LTD. Japan) and drying at room temperature. As a final step, the powdered extract was stored at 4 °C until it was needed^28^. During this study, the prepared pomegranate extract was called P-Extract.

### Biosynthesis of nanoparticles

The silver nanoparticles were synthesized using the pomegranate extract (called P-AgNPs) by following the method previously described by Khorrami et al*.* with slight modifications^28^. Briefly, after dissolving 50 mg of the extract powder in 100 ml of deionized water, we adjusted the extract pH to 8 using NaOH (1 M). Fifty ml of AgNO_3_ aqueous solution (6 mM; Merck, Germany) was next gradually added to the mixture while vigorously stirring and the final combination was allowed to stir for 2 h at 40 °C. The final product was purified by centrifuging at 12,000 rpm for 10 min and washing the deposited AgNPs with deionized water.

### Physicochemical characterization

We used a JASCO V-670 UV-VIS-NIR spectrophotometer (Tokyo, Japan) to measure the UV–visible absorbance of the colloid of Ag nanoparticles as a primary characterization. In order to determine functional groups involved in Ag nanoparticle biosynthesis, KBr pellets of the extract and P-AgNPs were prepared and analyzed using Fourier transform infrared spectroscopy (FTIR; JASCO Ltd., Tokyo, Japan) in the wavenumber of 500–4000 cm^−1^. The Transmission Electron Microscopy (TEM; ZEISS LEO912-AB, Germany) method was used in order to examine the morphology and dispersity of Ag nanoparticles. Also, Ag nanoparticles synthesized in this study were tested for their surface charge and size at pH 7 and 25 °C using a Horiba SZ-100 Zetasizer instrument (Kyoto, Japan).

### Cytotoxicity assay (MTT assay)

The extract, Ag nanoparticles, and Meth were evaluated for their cytotoxic activity against dopaminergic human neuroblastoma cells (SH-SY5Y) using MTT colorimetric assays. The cell line was purchased from the Pasteur Institute, Tehran, Iran. Briefly, A 96-well plate was cultured with 6000 cells/well in DMEM high glucose medium with 10% FBS, and 1% penicillin–streptomycin antibiotic (Biosera, England) for 24 h at 37 °C, 5% CO_2_, and 95% humidity. Then, 100 μl of fresh medium containing different concentrations of the extract (8.5, 17, 25, 35, and 50 µg/ml), PAgNPs (8.5, 17, 25, 35, and 50 µg/ml), and Meth (0.25, 0.5, 0.75, 1.5, 2, 2.5, 3.5, 7 µg/ml) was replaced with the old one, followed by 24 h incubation. Following drainage and washing with PBS, the wells were refilled with 100 µl of medium with 10% MTT (5 mg/ml; Sigma-Aldrich, Germany), and incubation was completed for a further 4 h. As the final step, after the removal of the MTT solution, each well was filled with 100 ml of Dimethyl sulfoxide (DMSO), and the plate was stored at 37 °C for 10 min. As a control, cells that were not treated and cultured under the same conditions were considered. An ELX800 spectrophotometer (BioTek, USA) was used to measure the absorbance of the wells' contents at 570 nm. Based on Eq. (1), the viability percentage of the cells was calculated.

1 $$Cell \; viability (\%)=\frac{Absorbance \; of \; treated \; cells}{Absorbance\; of \;control \;cells}\times 100$$

### Protective effect evaluations

Having the cytotoxic concentrations of each agent determined, to evaluate the potential protective activity of the extract and AgNPs against the cell death induced by Meth, the cells were pretreated with sub-toxic concentrations of the extract (3 µg/ml) and AgNPs (1.5 µg/ml) for 2 h and 4 h, followed by exposure to IC_70_ of Meth for 24 h.

### MTT assay

Here, the protective potential of the compounds was determined based on the viability of the pretreated cells compared to non-pretreated ones. Toward this end, the MTT assay was conducted as described in the previous section.

### ROS assay

To evaluate the protective activity of the extract and AgNPs on ROS production in SH-SY5 cells exposed to the Meth, we applied the slightly modified Wanga and Roper's method^51^. The first step was to seed 30,000 cells in each well of a 96-well plate and incubate them for 24 h. Afterward, the culture medium was replaced and cells were incubated for 24 h at 37 °C while treated with the extract or AgNPs (solved in 100 μl of fresh medium). After 4 h of incubation, the medium supplemented with Meth was added to each well, and incubation was continued for 24 h. As the final step, the content of the wells was replaced with fresh DMEM containing DCFH-DA (20 μM; Sigma Aldrich, Germany) and incubated for 1 h at 37 °C. A BioTek-FLX-808 (USA) fluorescent/ELISA reader was used to detect the fluorescence intensity (exciting wavelength: 485 nm; emission wavelength: 538 nm). Finally, the fluorescence intensity of the treated cells was compared with that of those in the control group (which did not receive any treatment).

### Thiobarbituric acid reactive substance (TBARS) assay (MDA assay)

Thiobarbituric acid reactive substance (TBARS) assay was used to evaluate the oxidative stress in the cells as previously described with slight modification^52^. To accomplish this, the cells were pretreated with P-AgNPs or the extract and subsequently exposed to Meth. After 24 h of incubation, they were detached with trypsin, washed, and resuspended in 10 ml of PBS. Next, 1 ml of thiobarbituric acid (TBA) reagent containing 0.375% 2-TBA, 15% TBA, and 0.25 N HCl was added to the cell suspension. The samples were then heated at 95 °C for 20 min, flowed by chilling to room temperature and centrifuged at 10,000×*g* for 10 min. After that, the TBA method was used to measure TBA-reactive substances formed by lipid peroxidation at 535 nm. Untreated cells and the cells treated with Meth were considered control. Malondialdehyde (MDA) level (ng/mg) was reported as the final result.

### Mitochondrial membrane potential assay (MMP or ΔΨm)

The effect of the extract and nanoparticles pretreatment on the mitochondrial membrane potential of cells exposed to the Meth was determined with the Rhodamine 123 (RH-123; Sigma Aldrich, Germany) probe and fluorescence spectrophotometry. Briefly, the cells were incubated with 10 µM of RH-123 in the dark for 30 min at 37 °C. After the incubation, the cells were washed (three times) with PBS and analyzed immediately on the fluorescence plate reader (BioTek, USA). An emission wavelength of 538 nm and an excitation wavelength of 485 nm was used to quantify cells' fluorescence intensity. A fluorescence percentage of control cells was used to express the results^53^.

### DNA fragmentation assessment

To evaluate the effect of the Meth and the protective agents on the cells’ DNA, 10^6^/well of the cells were seeded in 6-well plates and pretreated with the P-extract or P-AgNPs for 4 h; the cells were then exposed to the Meth for 24 h. The cells were harvested by scraping in 1 mL of PBS and lysed in 500 µL of lysis buffer for one hour at 55 °C. The extraction of DNA was performed by phenol/chloroform/isoamyl alcohol (25:24:1 v/v/v), precipitating it with ethanol, and resuspending it in Tris–EDTA buffer containing 20 g/mL RNase. The DNA content was quantitatively analyzed, by loading an equal amount of DNA on a 1.0% agarose gel containing 1 μg/mL ethidium bromide. Exposing the gel to ultraviolet light, the DNA fragments were visualized and imaged. The cells grown on the culture medium only were considered control^54^.

### Gene expression assay (real-time PCR)

To study how the protective compounds affect the expression of genes involved in the cell death process, the real-time polymerase chain reaction (RT-PCR) technique was applied. Briefly, SH-SY5 cells were firstly cultured in a 6 cm plate, and about 24 h later, when their confluency reached 80%, a fresh medium containing Ag nanoparticles (1.5 µg/ml) and the extract (3 µg/ml) were substituted for the culture medium. The plates were incubated for 4 h before adding Meth to the wells. After 24 h of incubation, the relative expression of genes listed in Table 1 was measured in treated cells compared to untreated ones (control group). The 2 × universal SYBR green master mix (BioFACT™, South Korea) was utilized in the RT-PCR process and each measurement was done in duplicate. As previously described in detail^55^, an amplification program, including a denaturation step (15 min) and 40 cycles of amplifications was run using a Bio-Rad iQ5 detection system (Bio-Rad, Richmond, CA, USA). In addition to melting curve analysis, PCR products were also electrophoresed on an agarose gel (1%) in order to determine their amplification specificity. PCR data were normalized with the Beta 2 Microglobulin (B2Ma) universal housekeeping gene and analyzed using the 2^−ΔΔCT^ ratio. Table 1 lists the sequences, lengths, and NCBI accession numbers of the primers.

**Table 1:** Details of the primers utilized in RT-PCR analysis.

Primer name	Primer sequence	PCR product size (bp)	NCBI accession number
TNF-α	F: ACCAGCAGATGGGCTGTACCTTAT	107	M_012675.3
	R: ATGAAATGGCAAATCGGCTGACGG		
P53	F: GCCCCTCCTCAGCATCTTAT	242	NM-001126118.2
	R: CTGTTCCGTCCCAGTAGATT		
PI3K	F: TTTAAACGCGAAGGCAACGA	101	NM_008760659.3
	R: AGTCTCCTCCTGCTGTCGAT		
AKT	F: CGTGTGGCAGCACGTGTACGAG	201	NM-001382431.1
	R: CCGCTGGCCGAGTAGGAGAAC		
NF-κB	F: AGAGCAACCGAAACAGAGAGG	227	NM_001276711.1
	R: ATATGCCGTCCTCACAGTGC		
PTEN	F: AGGGACGAACTGGTGTAATGA	100	NM_000314.8
	R: CTGGTCCTTACTTCCCCATAGAA		
B2M	F: GCTCGCGCTACTCTCTCTTT	134	NM_004048.4
	R: CGGATGGATGAAACCCAGACA		
BAX	F: CCCGAGAGGTCTTTTTCCGAG	155	NM_001291430.2
	R: CCAGCCCATGATGGTTCTGAT		
BCL-2	F: CATGTGTGTGGAGAGCGTCAA	88	NM_000657.3
	R: GCCGGTTCAGGTACTCAGTCA		
Cyt C	F: CCCAAAGGGAGCTTCAGG	108	NM_001862.3
	R: CGACGCTGGTATTGTCCTCT		
Cas 3	F: TGGTTCATCCAGTCGCTTTG	101	XM_047416239.1
	R: CATTCTGTTGCCACCTTTCG		

### AnnexinV/PI apoptosis assay (flow cytometry)

Fluorescein isothiocyanate (FITC) and Propidium Iodide (PI)-conjugated annexin V intercalating agents were used to stain the cells and determine the apoptosis/necrosis rate of SH-SY5 cells. Briefly, SH-SY5 cells (2 × 10^5^) were treated with Meth for 24 h following a 4-h pretreatment with the indicated concentration of the extract or AgNPs. After detachment of the incubated cells, they were resuspended in 100 μL of 1 × binding buffer, including 20 μg/ml of Annexin V and 1 μg/mL of PI for 15 min at RT. These cells were finally assessed on a BD FACSVerse (CyFlow Space, Sysmex, Germany).

### Statistical analysis

During the study, each test was conducted 3 times independently. Data were statistically analyzed based on the One-way analysis of variance and t-test using the SPSS software version 23. The differences with P values ˂ 0.05 were determined as significant. Results were expressed as the Mean ± SEM compared with the control group (Supplementary Information).

### Ethics approval and consent to participate

The pomegranate fruit was used in the present study according to the Herb Garden of the University of Isfahan guidelines.

## Supplementary Information

Supplementary Information.

## Data Availability

Data sharing is not applicable to this article as no datasets were generated or analysed during the current study.
