# Supplementary figures and images for: Neuroprotective activity of green synthesized silver nanoparticles against methamphetamine-induced cell death in human neuroblastoma SH-SY5Y cells
Source: Sci Rep. 2023 Jul 22;13:11867. doi: 10.1038/s41598-023-37917-0 (PMC10363122; doi:10.1038/s41598-023-37917-0)

**Supplementary**

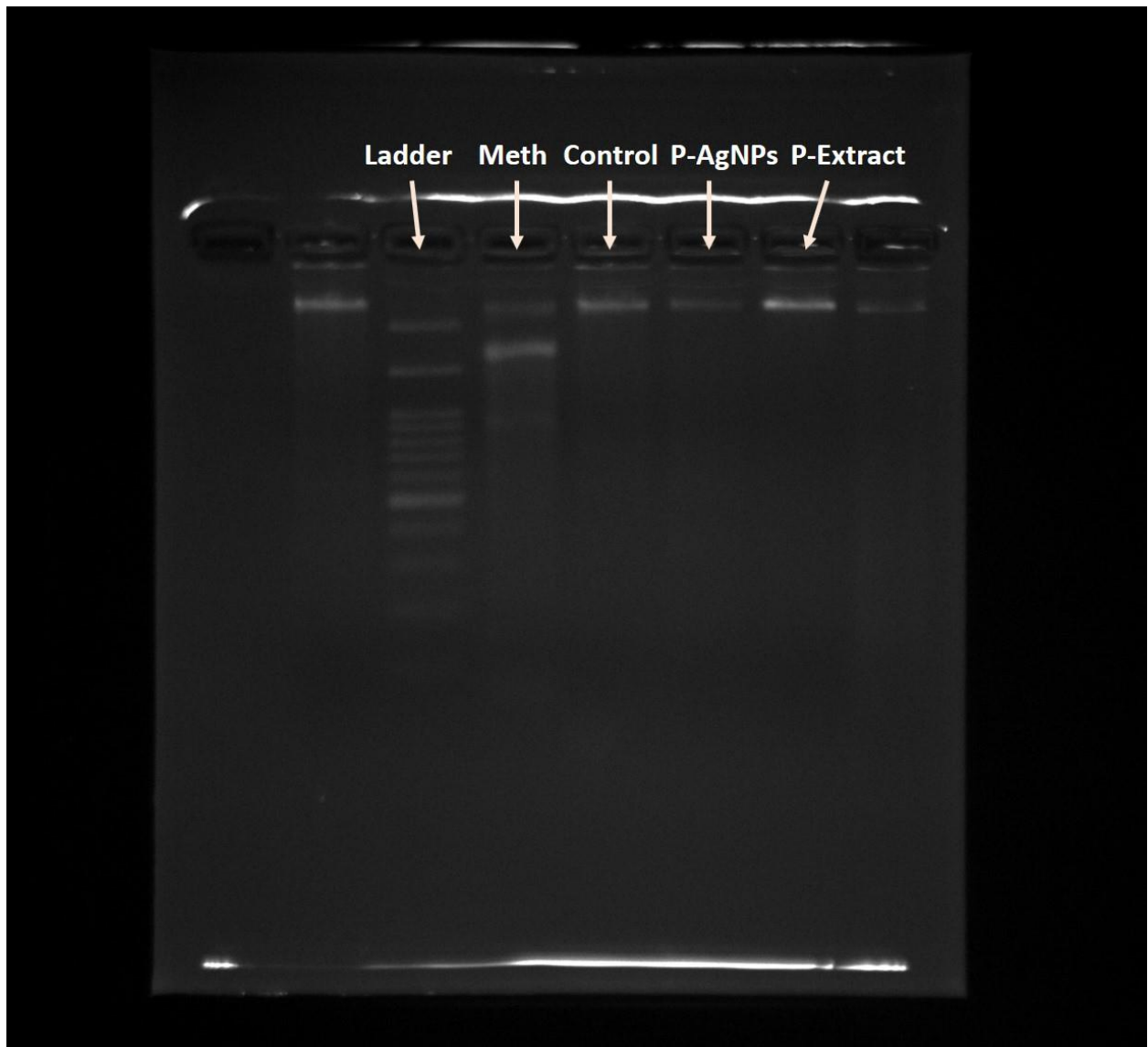

The whole gel related to Figure 7

Supplement: Supplementary file 1 — Supplementary Information. [file 41598_2023_37917_MOESM1_ESM.pdf]
